# Human and nonhuman norms: a dimensional framework

**DOI:** 10.1098/rstb.2023.0026

**Published:** 2024-03-11

**Authors:** Kristin Andrews, Simon Fitzpatrick, Evan Westra

**Affiliations:** ^1^ Department of Philosophy, York University, Toronto, ON, Canada M3J 1P3; ^2^ Department of Philosophy, John Carroll University, Cleveland, OH, USA; ^3^ Department of Philosophy, Purdue University, West Lafayette, IN, USA

**Keywords:** social norms, animal culture, animal cognition, norm psychology, punishment, teaching‌

## Abstract

Human communities teem with a variety of social norms. In order to change unjust and harmful social norms, it is crucial to identify the psychological processes that give rise to them. Most researchers take it for granted that social norms are uniquely human. By contrast, we approach this matter from a comparative perspective, leveraging recent research on animal social behaviour. While there is currently only suggestive evidence for norms in nonhuman communities, we argue that human social norms are likely produced by a wide range of mechanisms, many of which we share with nonhuman animals. Approaching this variability from a comparative perspective can help norm researchers expand and reframe the range of hypotheses they test when attempting to understand the causes of socially normative behaviours in humans. First, we diagnose some of the theoretical obstacles to developing a comparative science of social norms, and offer a few basic constructs and distinctions to help norm researchers overcome these obstacles. Then we develop a six-dimensional model of the psychological and social factors that contribute to variability in both human and potential nonhuman norms.

This article is part of the theme issue ‘Social norm change: drivers and consequences’.

## 1. Barriers to a comparative science of norms

Nearly every aspect of human life is shaped by social norms, the ‘often informal rules that structure human behaviour, regulating what is appropriate, required, prohibited or permitted’ ([1], p. 36). This makes understanding the cognitive and evolutionary underpinnings of social norms critical to predicting, explaining and intervening upon human social behaviour. In recent years, a growing number of authors have approached the cognitive science of norms from a comparative perspective, with some suggesting that many animal communities, from nonhuman primates to eusocial insects, are also governed by social norms (table 1; [2,5,6,22–31]). These proposals have proven controversial, as the capacity for social norms is frequently cited by other researchers as uniquely human, part of a suite of cognitive and evolutionary adaptations that contribute to ‘the secret of our success' [32–37]. Adjudicating between these competing claims is challenging for two reasons: first, there is considerable conceptual confusion surrounding the explanatory targets of the cognitive science of social norms, such that importantly different senses of ‘social norm’ are regularly conflated; second, there is no consensus about the nature of the psychological capacities underlying human norm psychology, and so there is no agreed-upon benchmark against which nonhuman animal performance might be measured [38]. This paper offers a framework for moving past this impasse and towards a more fruitful comparative science of social norms.

**Table 1.** A non-exhaustive list of examples of potential social norms in animals. As argued in the text, key to establishing whether these are social norms is identifying mechanisms of social maintenance.

domain	taxa	potential social norms
Treatment of infants and juveniles	Chimpanzees, dolphins, killer whales, sperm whales	Chimpanzee infants enjoy greater tolerance from adults, including high-ranking males [2].
		Reports of third-party protest and intervention in attacks on in-group chimpanzee infants [3–5].
		Captive chimpanzees looked longer at videos of infanticide than videos of aggression between adults [6].
		Dolphins and killer whales help others deliver infants and help raise newborns to surface [7–9].
		Sperm whale females take turns babysitting each other's calves while mothers dive to hunt [9].
Play interactions	Domestic dogs, chimpanzees	Characteristic signalling to initiate and maintain play intent (e.g. play bow in dogs). Handicapping when playing with weaker partner [10,11].
Dominance hierarchies	Chimpanzees, baboons, African elephants, multiple species of bee, wasp and ant	Dominance hierarchies in chimpanzees determine who gets to do what, when, and with whom [2].
		Socially maintained ‘pacific culture’ with more relaxed dominance hierarchy in a troop of baboons [12].
		Older bull African elephants restrain the aggressive behaviours of younger males. Culling of adult males leads to aberrant aggressive behaviour and breakdown of social structures [13,14].
		‘Enforced’ worker altruism in several species of bee, wasp and ant: workers forgo reproduction and worker egg-laying is policed by nestmates [15–17].
Costly cultural conformity	Vervet monkeys, chimpanzees, killer whales	Vervet monkey groups induced with different preferences for blue- and pink-coloured corn; infants acquired the preference of their natal group, but then switched when moved into a group with the opposite preference, despite being provisioned with both colours [18].
		Immigrant chimpanzees switch from tool material preference of natal group to sometimes less efficient material of new group [19].
		Immigrant chimpanzee who adopted local female-specific tradition (cross-arm walk) found to be better integrated into community than immigrant who did not adopt the tradition [20].
		Persistent maladaptive behaviour in killer whales: the southern resident population specializes in feeding on declining stocks of chinook salmon, despite availability of other species [9,21].

First, it is important for norms researchers to clearly distinguish between the *group-level behavioural regularities* that comprise social norms (e.g. a group of individuals queuing up at a bus stop) and the *psychological processes* that lead individuals to participate in these regularities (e.g. a given person's belief that queuing is obligatory). We will refer to the former as *normative regularities* and the latter as *norm psychology*. This distinction matters, because the basic goal of the cognitive science of social norms is to explain normative regularities by developing models of norm psychology, which can then be deployed for a variety of other practical purposes, such as norm change.

Much traditional social norm literature has focused on developing models of norm psychology (e.g. [39–42]) and the roles that it has played human evolution, such as in facilitating large-scale cooperation (e.g. [43–45]). The sheer variety of mechanisms identified in this literature is striking, ranging from explicit, discursive representations of rules [33,34], to distinctively social affective processes [46], to richly metacognitive representations of other people's beliefs [39], to mechanisms underlying skilled action control [47,48], to a capacity for shared intentionally [35], to a domain-specific ‘norm system’ shaped by gene–culture coevolution [42,45], to phylogenically widespread domain-general mechanisms for association and reinforcement learning [49,50]. Notably, many of these proposals are not mutually exclusive and all of them plausibly explain some human social norms. This has led some authors to argue for pluralistic models of human norm psychology, constituted by a variety of different cognitive and affective processes that operate differently in different contexts [38,50].

In contrast to the rich literature on models of norm psychology, normative regularities themselves are currently undertheorized, with most definitions of social norms conflating the latter with the former: according to this approach, normative regularities are just those group-level patterns of behaviour that are caused by individual norm psychologies. But this seems to presuppose precisely what the cognitive science of norms is supposed to explain—namely, where normative regularities come from. The conflation of norm psychology and normative regularities is not always noticeable in human cases, where there is an intuitive consensus about which behaviours will count as social norms; thus, norms researchers may disagree about what causes people to queue, but they will all agree that queuing is a paradigmatic norm. However, it becomes especially problematic in debates about whether or not nonhuman animals have social norms. When a dominant chimpanzee intervenes in a conflict between two subordinates, is this evidence of a social norm being enforced [5]? Most researchers will reply that this depends upon whether or not this behaviour was produced by some homologue of human norm psychology. However, this approach effectively presupposes that we have a good sense of what *human* norm psychology consists in. But since there is no consensus about which particular mechanisms are constitutive of human norm psychology, there can be no consensus about whether this behaviour is in fact an instance of a normative regularity. This creates an unfortunate barrier to comparative and evolutionary research on this topic.

There is an alternative, however: rather than defining them in terms of norm psychology, it is possible to characterize normative regularities in a minimally psychological way that presupposes very little about their underlying mechanisms and instead focuses on readily operationalized patterns of behaviour. This approach would permit researchers to *first* identify normative regularities out in the world and *then* develop a bottom-up taxonomy of all the psychological processes that bring them about. This is the approach that we take in the following section.

## 2. Normative regularities

Following Westra & Andrews [38], we define normative regularities as *socially maintained patterns of behavioural conformity within a community*. There are three key notions within this definition: *patterns of behavioural conformity, social maintenance* and *community*.

With *patterns of behavioural conformity*, we aim to capture the descriptive, statistical element of social norms. Social norms reflect how things are done in a given community: ant queens lay eggs, workers don't. Dogs signal friendly intent with a play bow. One community of chimpanzees holds hands when they groom, another holds their partner's elbow. One community of humans greets with a handshake, another with a bow.

Importantly, this use of ‘conformity’ does not make assumptions about the underlying motivations or mechanisms that explain individual adherence to the pattern. Thus, it should not be conflated with more specific notions of conformity, such as *Aschian conformity* (copying others' behaviour despite having countervailing evidence or preferences; [51]) or *conformity bias* (a disproportionate tendency to copy the most common behavioural variant in the local population; [52]). Here, ‘conformity’ simply refers to patterns of similar behaviours that are statistically common in a population. See Whiten [53] and van Leeuwen *et al*. [54] for helpful discussions of these different ideas about conformity.

Of course, not all patterns of behavioural conformity are normative regularities. When groups of humans drink lots of water on a hot day, we do not explain this in terms of social norms, but rather in terms of shared environmental conditions and biological needs. In prototypical social norms, the pattern in question is shaped by *social incentives* that motivate agents to conform and deter them from nonconformity. Often, norm theorists characterize such social incentives in terms of *punishment* or *sanction* [43,55]. Our concept of *social maintenance* is meant to capture this aspect of norms. However, it casts a wider net than the traditional focus on punishment, and includes positive as well as negative social incentives, such as social inclusion and gains in status that are also important for sustaining social norms [38].

Finally, by ‘*community’* we are simply referring to the target group, however it is defined. Bicchieri [39] calls this the ‘reference class’: the group to which the behavioural pattern is indexed. This may include all members of a population, a dyad (norms can be set up within pairs of individuals, such as partners, friends, and parent and offspring) or other sub-group, or it may be species universal and include all conspecifics, depending on the target behavioural pattern.

This definition aims to cover examples of prototypical human social norms, such as norms of dress, etiquette and morality. With respect to the examples of potential nonhuman social norms listed in table 1 that have been discussed in the literature, a key open question is whether there is good evidence for social maintenance of the relevant behavioural regularities. This is central to determining whether they should be considered normative regularities on our approach. For instance, examples of costly conformity to cultural practices, where individual animals follow a group tradition—such as favouring a particular tool material or foraging strategy—at some cost (e.g. an immigrant switching from a more efficient strategy to a less efficient one, or foregoing other productive opportunities) have been cited as potential social norms [19,31]. However, what is generally lacking is information about what happens when individuals deviate from or conform to the behavioural regularity—e.g. are there corresponding social costs to nonconformity and rewards for conformity? This is where we believe future comparative research on social norms should be directed: identifying robust patterns of behavioural conformity and mechanisms of social maintenance.

Some critics might object that our characterization of normative regularities is overly broad and that too many nonhuman behaviours would fall within its scope. For instance, Mormon crickets demonstrate a kind of social maintenance during swarming events when they gather and march, eating all the nutrients in their way. Insects that deviate from this pattern are cannibalized by their swarm-mates, placing them in a march-or-die situation [56]. Since swarming is a kind of normative regularity, one might worry that the construct is too permissive, especially given that such self-organizing regularities are common—in insect behaviour in particular [57].

However, the permissiveness of our definition is intentional. Inevitably, how normative regularities are initially characterized will impact the scope of the ensuing comparative investigation: the more permissive we make our definition, the more potential nonhuman social norms it will cover, resulting in a model of norm psychology comprising a wider range of underlying mechanisms. The more restrictive we make our definition, the more we potentially restrict the range of mechanisms under consideration. Given the wide range of psychological mechanisms that have already been proposed to underlie human norm psychology, initially erring on the side of permissiveness is appropriate. This still leaves room for the future addition of more restrictive characterizations of normative regularities, but these should be based on bottom-up, evidence-driven considerations, rather than *a priori* stipulation.

As the Mormon cricket example shows, normative regularities can potentially be realized under a wide range of conditions and be supported by many different psychological mechanisms. This changes the way we must think about constructing our model—or rather, *models—*of norm psychology. Instead of just assuming that norm psychology is a unified or homogeneous system that gives rise to normative regularities, we must instead ask: what sorts of psychological processes are likely to give rise to normative regularities? The short answer is: *quite a few*. This provides norm researchers with an opportunity to develop a more fine-grained, pluralistic way of thinking about the psychology of social norms and the potential variability that might underlie it.

In the sections that follow, we identify four key dimensions of psychological variability that are likely implicated at the individual level in both human and potential nonhuman normative regularities, each along increasing levels of cognitive and interactive complexity: *rule-following, behavioural understanding, collective agency* and *motivation.* We also include two further dimensions of variability corresponding to different forms of social interaction, which support social maintenance and thereby bridge the gap between norm psychology and normative regularities: *pedagogy* and *punishment*.

**Figure 1.**
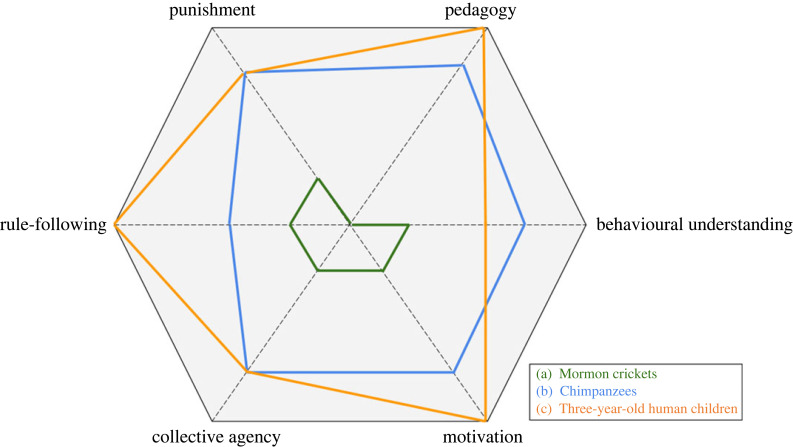
Geometrical representation of the social norms-space in three species. Human children are chosen to illustrate that this space can also be expected to change over development. This space can also be used to represent particular norms and the processes that underpin them.

The dimensional approach enables researchers to graphically represent ‘normative profiles’ for different norms, different communities and different species. These profiles capture multiple dimensions of complexity in both the psychological and social processes that characterize the normative behaviours of different species, and highlight the different combinations of processes that may give rise to normative regularities both within and across species. We expect that future research will find that the gradients of the norm dimensions cluster in interesting ways, offering a novel approach to categorizing social norms given their particular geometries. Figure 1*a–c* depicts hypothetical normative profiles for Mormon crickets, chimpanzees and human three-year-olds based on existing research. Note that this way of representing normative profiles should not be taken to imply any kind of evolutionary hierarchy. It is rather a tool that represents the space of possible norms for different species and different communities within these species.

## 3. Dimensions of norm psychology

### (a) Rule-following

Intuitively, social norms have something to do with rules [55]. However, it is in principle possible to describe the same observed behaviour in a way that is consistent with infinitely many possible action-guiding rules [58,59], making this feature of norms difficult to map onto underlying cognitive processes. Many accounts of the psychology of norms therefore distinguish between *following* a rule and merely *acting in accordance with* a rule, where genuine rule-following requires some kind of rule-based *representations* [24,25,33,34,60,61]. However, what this amounts to in practice is not always clear. Most theorists acknowledge that many of the norms we follow are never explicitly articulated, but rather guide our behaviour in a more tacit manner (consider, for example, the unspoken and culturally variable norms that govern personal space). Daston [62] also draws a distinction between rules as algorithms that provide explicit step-by-step instruction and rules as models or paradigms to emulate, such as a monk following the example of Saint Benedict. The *rule-following* dimension of normative regularities captures this variability.

#### (i) Level 1: Emergent regularities

Rule-following behaviours that involve very little mental representation, with rule-like patterns of behaviour emerging organically from individuals' shared biology. In the Mormon cricket case, the regularity is driven by basic mechanisms for predation and evading predators.

#### (ii) Level 2: Implicit representations

Rule-following behaviours that are driven by relatively simple cognitive processes, such as model-free reinforcement learning. Such mechanisms store and update information about the regularity in question without representing them explicitly. These mechanisms are plausibly instantiated in a wide range of taxa, and likely explain many apparent animal normative regularities. Some predictive processing models of cognition also permit implicit representations of norms as clusters of predictions about the behaviours of others [46].

#### (iii) Level 3: Non-propositional representations

Rule-following behaviours that are supported by non-propositional representations such as cognitive maps or embodied motor plans, where standards of correctness are instantiated by affect-based guidance systems. Birch [47] argues that model-based control mechanisms originally evolved in the hominin lineage in the context of standardized toolmaking, and were exapted for a broader range of social practices. However, Andrews & Westra [23] argue that this kind of representational mechanism is likely to be phylogenetically widespread.

#### (iv) Level 4: Propositional representations of rules

Rule-following behaviours supported by representations with distinctively propositional characteristics such as compositionality, generality and recursion. Whether or not any nonhuman animals are capable of such forms of representation is a matter of controversy [63,64]. One potential example of an animal normative regularity supported by this kind of representation is baboons’ representations of dominance hierarchies, which Cheney & Seyfarth [65] have argued possess many of the features of a language of thought.

#### (v) Level 5: Public linguistic representations of rules

Rule-following behaviours supported by language that facilitates public discourse around rules (what Heyes [50] calls ‘norm commentary’). Such linguistic representations are likely unique to human beings.

### (b) Behavioural understanding

Some authors model the acquisition of norms as a mentalistic process involving complex inferences about other agents' mental states. Famously, Lewis's [66] analysis of social conventions required all parties be able to represent each other's mutual assent to a given behavioural regularity as *common knowledge*—that is, that all parties know that P, all parties know that all parties know that P, all parties know that all parties know that all parties know that all parties know that P, and so on. This kind of recursive mental-state attribution is notoriously difficult, even for adult humans [67,68]. Bicchieri's model of norms implicates less elaborate but still quite sophisticated mentalizing abilities, specifically, belief-attribution [39,40]. According to her account, a social norm is a behavioural rule that individuals prefer to follow provided they believe that: (a) most people in their reference network conform to it (empirical expectation) and (b) most people in their reference network believe they ought to conform to it (normative expectation) [39,40]. Other theorists invoke mentalizing in other aspects of normative cognition, including punishment [69], learning from evaluative feedback [70] and in the affective predictions that inform how it might feel to violate a norm [46].

One challenge with heavily mentalistic approaches to the psychology of norms is that they risk over-intellectualizing everyday human norm-conformity and unnecessarily excluding related nonhuman behaviours in the process [22,25]. For example, Bicchieri's account requires an ability that most developmental psychologists believe emerges only by four-and-a-half years of age in humans [71], and whose use in everyday social cognition is a matter of considerable disagreement [72,73]. Whether or not great apes or any other nonhuman animals are capable of belief-attribution is even more controversial [72,74–77]. In a critique of Bicchieri's account, Andrews [22] argues that in this model the real function of belief-attribution is behavioural prediction and interpretation, and that there are many alternative cognitive mechanisms for behavioural prediction and interpretation that do not require belief-attribution, such as generalizing from the situation, or from an individual's past behaviour [72]. While sophisticated mentalistic abilities may be implicated in some human norms, other human norms are likely supported by simpler mentalistic and nonmentalistic processes.

#### (i) Level 1: Submentalistic behaviour understanding

The capacity to anticipate and learn new behaviours via low-level processes that represent only surface-level, nonmentalistic properties of behaviour, including associative learning and reinforcement learning. Several authors have argued that such capacities are implicated in the psychology of human norms [38,49,50]. For example, associative learning processes supporting behavioural imitation [50] might lead novices to mimic the norm-governed behaviours of more mature conspecifics. Processes of habituation might support the gradual automatization of norm-conforming behaviours over time [78]. Processes like these are widely distributed among nonhuman animal species [79].

#### (ii) Level 2: Minimal mentalizing

The capacity to predict and interpret behaviour using representations of non-representational and/or non-propositional mental states. We use this term as a catchall that encompasses a range of proposals about the kinds of mentalizing abilities that phylogenetically or ontogenetically precede the emergence of metarepresentational mindreading (e.g. [72,80–83]). Among these capacities, the ability to identify goal-directed or intentional actions is particularly relevant to distinguishing actions that conform to norms from actions that are accidental or haphazard. Identifying knowledge and ignorance is also likely important in determining from whom one should learn a norm. Both of these capacities are thought to be widespread among nonhuman animals [84].

#### (iii) Level 3: Metarepresentational mentalizing

The capacity to predict and interpret behaviour using representations of propositionally structured, causally efficacious mental states whose contents can diverge from reality, especially via the concept of *belief*. The capacity is most commonly measured using some version of the false-belief task, which aims to establish whether a participant can grasp when another agent has misrepresented the world [85]. Belief-attribution is plausibly implicated in some strategies for learning social norms, such as when one ascertains the presence of a social rule on the basis of an inference about what other agents believe the rule to be and whether it ought to be followed [39,40]. This belief-based form of norm learning is implausible in cases of norm-acquisition in young children for whom such forms of belief-attribution are either absent or highly fragile (e.g. [86]). While there is evidence that some nonhuman primates are capable of belief-attribution [74,75], it is unknown whether this ability is implicated in any nonhuman cases of norm-learning.

#### (iv) Level 4: Recursive mentalizing

The capacity to recursively embed representations of mental states as part of the content of another mental-state attribution, as in *A believes that [B believes that [C believes that [P]]].* In theory, this capacity might be implicated in the initial resolution of coordination problems (e.g. whether everyone should drive on the right or left side of the road); once such a solution has been achieved, it may become the basis for a normative regularity. There is no evidence that this capacity is present in nonhuman animals. Given the cognitive demands that this kind of reasoning seems to pose even for mature humans, it may be a relatively rare means of establishing norms.

### (c) Collective agency

Some theories of the psychology of norms require representations of collective group membership. The idea is that norm understanding is understanding of what the collective ‘*we*’ expects of each community member [37]. For example, Bicchieri's account delimits the conditional social preferences undergirding social norms to members of a contextually defined reference network, implying a fairly advanced capacity for mentalizing. Tomasello's [35] account, meanwhile, emphasizes a distinctively collective way of representing norm-conformity derived from a more basic capacity of shared intentionality. However, other accounts of norm psychology do not assign any particular emphasis to how other parties to a norm are represented (e.g. [42,46,47,50]). This difference in emphasis leaves room for a range of ‘collective’ ways of representing co-participants in a normative regularity.

### (i) Level 1: Individual intentionality

Individuals acting only on their own goals and pursuing their own action plans. In such cases, other agents and their actions are merely incidental, and there is no sense in which they act ‘together.’ Nevertheless, given the right conditions, this might suffice to give rise to socially maintained patterns of behavioural conformity. This is likely the case in the Mormon cricket example, where the social properties of the behavioural regularity are driven entirely by individualistic goal-pursuit. Westra & Andrews [38] suggest that some human norms are also like this. For example, when many individuals try to use a crowded escalator and those who are standing still are forced to move forward or risk repeated bumps from those coming up behind them, everyone is incentivized to walk rather than stand still: a simple yet real normative regularity.

#### (ii) Level 2: Coordinated intentionality

Individuals representing other participants as independent parties whose contributions may be necessary for achieving the individual's goal in an activity that requires coordination. In such cases, the ultimate goal is individualistic in nature, while the collective nature of the activity is secondary and instrumental; individuals need have no understanding and are in no way motivated by the fact that their action is collective. This is how Tomasello has characterized some of the naturally occurring collective behaviours of great apes in the wild [87,88], such as the group hunts of the Taï Forest chimpanzees, though Boesch [89,90] has defended a richer interpretation in terms of shared intentionality (i.e. Level 3 below).

#### (iii) Level 3: Shared intentionality

Individual representations of shared agency and the motivation to pursue joint goals *together*. In such cases, the social aspect of the activity is not a means to an end, but rather part of the content of the goal itself. According to Tomasello's [35] account of the ontogeny of norms, it is during episodes of joint goal pursuit that children begin to develop a sense of social obligation, based on the motivation to play their part in collective actions. While Tomasello has argued that this capacity to enter a shared ‘*we*-mode’—and by extension, normative cognition—is uniquely human, others have argued for the presence of key elements of shared intentionality in great apes [91,92] and domestic dogs [93]. Moreover, given the multiplicity of types of joint action and joint commitments in human and nonhuman cooperation, it is likely that there exists a complex continuum of feelings of mutual obligation from Level 2 to 3 [94].

#### (iv) Level 4: Social identity

Individual representations of a shared social identity. These are more temporally stable than shared intentionality and extend to the broader social group, while also tracing the boundary between ingroup and outgroup. This sense of a group ‘*we*’ is often invoked in the psychology of norms, particularly when norms are described in terms of ‘what *we* do around here’ [35]. Some theories of social identity see it playing an important intrapsychic, existential function for individuals, and see norm-conformity and enforcement as a means of reinforcing that function [95]. Where norm-conformity is co-extensive with group identity, it can also serve as a way of identifying potential cooperative partners, which in turn is argued to support processes like cultural group selection [96]. While it's unlikely that representations of social identity in the sense of Tajfel [95] exist in nonhuman animals, many species distinguish between ingroup and outgroup members [97–99], and many putative social norms closely track these boundaries (e.g. [19]; see [22] for a discussion).

### (d) Motivation

Another important source of variation in the psychology of norms is the motivation to conform to and enforce normative regularities. Some accounts emphasize the internalized nature of normative motivation [1], drawing a sharp contrast with instrumentally motivated behaviours. However, norm-conformity can emerge from a wide range of motivations that defy a simple two-way distinction, which is described by the motivational dimension in our model. We begin with simple instrumental motivations where norm-conformity is driven by immediate reward-seeking behaviour, then move through forms of motivation where the external rewards are increasingly remote and socially mediated, until they are removed altogether and replaced by endogenous rewards.

Two caveats are in order. First, this dimension does not necessarily map onto increasing levels of cognitive complexity: Level 2, for example, may require more cognitively taxing forms of actuarial intelligence than Levels 3 and 4. Rather, it tracks the extent to which a given behaviour is *directly* explained by external reward-seeking. Second, many behaviours are driven by multiple, mixed motivations: for example, one may follow a norm on the basis of self-interest *and* because of an intrinsic motivation. It is possible that several different motivational processes causally contribute to a given episode of norm-conformity or norm enforcement.

#### (i) Level 1: Basic motivation

An individual may conform to or enforce normative regularities because doing so is a direct means to pursuing their immediate self-interest or a result of biologically basic processes. For example, Wheeler [100] argues that capuchin monkey alarm calls in response to felids are likely driven by selfish motivations, with the aim of recruiting other individuals for the purpose of mobbing the predator.

#### (ii) Level 2: Reciprocal motivation

An individual may conform to a normative regularity not because it achieves some immediate goal, but because it is part of a reciprocally altruistic relationship with another individual and will yield some comparable benefit in return. In these cases, we should expect the persistence of the behaviour in question to be contingent upon continued reciprocation from a partner. Examples of reciprocally motivated adherence to behavioural regularities are widespread in nonhuman animals, include grooming behaviours in wild chimpanzees [101] and Barbary macaques [102], mobbing behaviours in pied flycatchers [103], as well as helping behaviours in captive Norway rats [104,105].

#### (iii) Level 3: Reputational motivation

An individual may conform to or enforce a normative regularity because they are motivated by reputational concerns [106,107]. A key behavioural signature of reputational motivations is that the conformity to the regularity in question varies depending on whether the animal is being observed by third parties. Evidence of reputationally motivated behaviours is scarce in nonhuman animals, but one clear example comes from cleaner fish, which learn to behave more cooperatively and refrain from defection when they are being observed by ‘eavesdropping’ client fish [108], and who have been found to cheat more when their conspecific partner cannot see them [109].

#### (iv) Level 4: Intrinsic motivation

An individual may conform to or enforce a normative regularity because they are intrinsically motivated to do so, absent any expectation of reputational benefit, future reciprocity, or immediate reward. A key behavioural signature of intrinsic motivation is the performance of unnecessary or otherwise costly actions that conform with a normative regularity even in the absence of an audience. Several accounts have posited mechanisms that might explain such intrinsically motivated behaviours, such as reinforcement learning [49], affect-based prediction-error minimization [46], complex prosocial emotions like guilt [110] and domain-specific affective adaptations for normative conformity [1,45]. While some of these mechanisms may be uniquely human (e.g. emotions like guilt), others (like reinforcement learning) are quite low-level and phylogenetically widespread. Intrinsic, endogenous motivations supporting norms in areas such as the treatment of infants may be shared across a wide range of taxa.

## 4. Interactive dimensions of variability

While the interactive features of norms can differ in many ways, two are particularly relevant for diagnosing normative regularities because they concern different ways in which norms can be socially maintained: punishment and pedagogy.

### (a) Punishment

Evolutionary anthropologists generally hold that third-party punishment played an important role in the evolution of human norm psychology by stabilizing cooperative norms at the community level [43,45]. Experimental work with economic games also suggests that humans are ‘altruistic’ punishers: willing to cooperate with others and punish norm violators at personal cost, even when costs are unlikely to be recouped [44,111]. This leads some philosophical models of norm psychology to take intrinsic motivation to punish norm violators as a key feature of such a psychology [42,112]). Moreover, because responses from affected parties can often be explained without appeal to ‘altruistic’ or ‘normative’ motivations, some theorists regard third-party punishment as essential to the definition of social norms, and doubts about the existence of third-party punishment in animals have motivated skepticism about animal norms [29,37,113].

There is, however, considerable disagreement about the extent and motivations behind human punishment—in particular, whether humans in non-WEIRD societies and outside the constrained context of economic games are altruistic punishers or routinely punish as third parties [114–118]. As with the motivational dimension, it is important not to conflate the question of whether normative regularities exist in a community with *how* they are enforced and the motivations behind enforcing them.

The classic biological definition of punishment requires that a punisher pay a cost to remove some benefit from the target [119], but the human norm literature adopts a broader understanding. For example, Kelly and Setman write that ‘Enforcement and punishment are broad categories, and can include correcting, withholding cooperation, communicating disapproval through body language or explicit criticism, ostracizing or gossiping about norm violators, or even physical violence’ ([55], s.1; see also [40]). This broader definition can accommodate cases of human punishment where punishers directly benefit, such as taking goods from the norm violator's family [120].

#### (i) Level 1. Second-party punishment

Violations are met with a negatively valenced response from the individuals directly impacted/harmed by the violation. Responses can include physical retaliation, a communicative signal of displeasure, a negative affective response, withdrawal from the violator, etc. Second-party punishment appears to be common in many species, especially in struggles for and maintaining dominance [17,119]. There is evidence that captive chimpanzees are willing to incur costs to watch the punishment of a human who behaved antisocially towards them [121] and willing to punish conspecifics who steal from them [113,122]. Other examples include cleaner fish clients punishing cleaners when they eat mucus rather than ectoparasites [123], brown-headed cowbirds (brood parasites) punishing egg-rejectors by destroying the entire nest [124] and female southern-masked weavers destroying inadequate nests built by courting males [125].

#### (ii) Level 2. Third-party punishment

Violations are met with a negatively valenced response by bystanders who are not directly impacted or involved in the transgression. In the laboratory, researchers have been unable to elicit third-party punishment in chimpanzees, operationalized as collapsing a food tray to prevent a thief from accessing stolen food [113]. There are reports of ‘policing’ in chimpanzees [5] and pigtailed macaques [126], understood as ‘impartial interventions by third parties in ongoing conflicts' (typically, the interveners are dominant individuals). When the definition of punishment is broadened to include partner choice behaviours, we also find evidence for punishment of chimpanzees who freeload in a cooperative task [127]. Vervet monkey females have been found to aggress towards males who fail to participate in intergroup fights, while males punish females who instigate intergroup fights [128,129]. Currently, the clearest evidence of nonhuman third-party punishment is in cleaner fish mutualism, where male cleaners chase away females who defect by eating the mucus rather than the ectoparasites of client fish [109,130].

#### (iii) Level 3. Institutionalized punishment

Violations are met with negative social maintenance via institutions that have been set up to manage violations. Key to institutionalized punishment is a many-against-one power structure [29]. These can include legal systems or coordinated responses by a police force, which for some theorists are the hallmark of a normative society and deemed to be human unique [34] (but see [16] for suggestions that it also exists in some eusocial insect societies).

### (b) Pedagogy

Normative regularities can also be enforced through acts of positive social maintenance that help naive individuals acquire norms to begin with, and that support knowledgeable individuals in correctly following the norm. Positive social maintenance behaviours can include teaching and facilitating social learning, praise, corrections and systems of institutionalized education [38]. In animals, active teaching has been operationalized as requiring effort on the part of a demonstrator that results in the learning of new information or skills on the part of the observer [131], and active teaching so-understood has been identified in several species [132]. However, active teaching appears to be rare and pedagogy more often takes the form of facilitative teaching, which involves modelling or tolerance of naive individuals. Thus, we can identify gradients of positive social maintenance from simple tolerance of social learners and apprenticeship learning, to the kinds of intentional teaching seen in human institutions.

#### (i) Level 1. Selective social toleration

Social learning is not sufficient for social maintenance, since social learning can be achieved without the demonstrator recognizing the existence of any learners. However, social learning that includes selective social toleration of some but not all other individuals in the group is a basic precondition for more active forms of pedagogy. Social toleration refers to the tendency to ‘be in proximity to conspecifics around valuable resources with little or no aggression’ ([133], p. 4). By emphasizing *selective* social toleration, we are focusing on who gets tolerated to the exclusion of others. Tolerance of social learners varies with social structure [134], and has been reported in chimpanzees that permit infants but not juveniles in the same learning context [2]. Both parenting and alloparenting may involve selective toleration of some learners over others, and in chimpanzees alloparenting has been found to be correlated with faster weaning, suggesting that these infants are learning to feed themselves more quickly [135]. We hypothesize that selective social toleration is a widespread form of social maintenance, since the cognitive demands on the demonstrator are low.

#### (ii) Level 2. Active feedback

Bystanders may facilitate another's acquisition of behavioural regularities by giving positive feedback to conformity, e.g. in terms of affiliative responses to conformity or partner choice—both likely key processes underlying norm maintenance in human beings. In animals, we see active feedback in female vervet monkeys selectively grooming males that participate in intergroup fights [128] and female cowbirds influencing the rate of acquisition of local song dialects by responding with sexual solicitation when male learners happen to produce local song elements [136].

#### (iii) Level 3. Active teaching/correction

Active teaching requires effort on the part of a demonstrator that results in the learning of new information or skills on the part of the observer [131]. Typical examples of active teaching in animals include meerkats that use task deconstruction when provisioning juvenile meerkats with scorpions [137], and tandem running in ants in which a knowledgeable ant takes a detour that allows the naive ant to observe landmarks [138]. Active teaching includes correction of errors by demonstrating the correct method in a slow or deliberate fashion. There is only anecdotal evidence of correction in animals, such as the observation of a mother chimpanzee intervening in her infant daughter's unsuccessful nut cracking by slowly demonstrating how to hold the hammer [139]. Corrections can also include acts of restorative justice, which is preferred in some small-scale human societies because it allows stakeholders to speak and be heard, offers compensation for losses and respects long-term relationships [140,141]. Third-party reconciliation was first reported in chimpanzees [142] and has been identified in a number of species, including domestic dogs ([143]; see also [144] on the ethology of peace).

#### (iv) Level 4: Institutionalized teaching

Some modern forms of human teaching take place in large, institutionalized communities under various levels of oversight and governance. While this potentially offers an efficient mode of norm transmission, its success depends upon reliable conformity to many other norms governing this kind of pedagogical context (e.g. the norm that pupils must be silent when the teacher is speaking). Following these norms is especially challenging for learners given the size of the group, which taxes their cognitive control and emotion-regulation abilities [47,145,146]. This makes institutionalized forms of teaching both socially and cognitively quite demanding; we expect it to be human-unique.

## 5. Conclusion

In this paper, we have advocated for a comparative science of social norms and offered a pluralistic, dimensional approach to the psychology of norms, showing how norms might emerge from a wide range of cognitive, affective and interactive processes, many of which we share with other animals. Researchers interested in understanding and changing norms in human communities should take a comparative approach to social norms more seriously than has been typical in the existing literature. It is impossible to fully understand any aspect of human behaviour or psychology without placing it in evolutionary and comparative context, yet most norm research focuses exclusively on humans. A comparative science of norms is thus essential to understanding the ways in which social norms may evolve and shape communities. However, comparative work on social norms has been held back by conflation between social norms as patterns of behaviour within communities (*normative regularities*) and the psychological mechanisms postulated to explain those patterns (*norm psychology*). To overcome this, we have offered a readily operationalizable characterization of normative regularities, which permits researchers to *first* identify norms out in the world and *then* develop a bottom-up taxonomy of the psychological processes that may bring them about.

As an initial step towards such a bottom-up taxonomy, we have offered a preliminary sketch of some of the different types of psychological mechanisms that may underpin and give rise to normative regularities. Our dimensional model offers norm researchers a tool for representing potential variability in norm psychology both within and across species, which we hope may be useful for fine-grained analyses of particular social norms of interest.

While further empirical research is necessary to determine how widely distributed normative regularities are in the animal kingdom, we emphasize the importance of addressing this question. Just as research into the distribution and psychological mechanisms supporting culture in animals has led to new ways of thinking about human culture and cultural cognition [76,147], we expect similar impacts from rigorous investigation of potential social norms in other species. Such research is also important for understanding animal communities themselves and human interactions with them. For example, it matters whether or not particular behavioural patterns that we find in animal communities, such as orangutans raiding fruit gardens, elephants destroying property, or street dogs attacking children, involve normative regularities that are socially maintained. If humans find such behaviours problematic and seek to change them, understanding how or whether they are socially maintained by members of the community is vital for designing effective and ethical interventions. If the behaviours are not socially maintained, and, say, just the product of individual or social learning, then attempts to adjust individual behaviour by providing alternative options or alternative models to copy may be effective. By contrast, if the behaviour is socially maintained by sanctions and/or social rewards within the community, then more of a group-level focus will be necessary.

Similarly, determining whether or not particular animal communities are governed by social norms and the psychological mechanisms that underpin them is important for understanding the impacts and potential ethical problems raised by other kinds of human interventions into animal societies. Be it culling African elephants, building green corridors to support migration, or managing captive communities, it is vital that we understand what social structures exist in the relevant societies, what mechanisms underlie them, and how they may be altered (or not) by the intervention [14]. For example, if elephant migration is governed by social norms about where and how the community migrates, elephants may be particularly resistant to adopting a new corridor, and theorizing about the mechanisms that give rise to such norms will be important for determining whether and how they might be encouraged to adopt it.

## Data Availability

This article has no additional data.
